# The unique effects of Covid-19 – A qualitative study of the factors that influence teachers’ acceptance and usage of digital tools

**DOI:** 10.1007/s10639-021-10574-4

**Published:** 2021-06-01

**Authors:** Olivia Wohlfart, Tim Trumler, Ingo Wagner

**Affiliations:** Center for Teacher Education, Karlsruhe Institute of Technology, Kaiserstraße 12, Geb. 20.52 (ZLB), 76131 Karlsruhe, Germany

**Keywords:** Technology acceptance model, Digitalization, Interview study, Pandemic, Secondary education

## Abstract

The objective of this study is to examine the factors that influence teachers’ acceptance of digital tools for undertaking distance teaching during the Covid-19 pandemic. Based on the variables of the technology acceptance model, we have conducted interviews with 15 secondary school teachers with varying degrees of professional experiences and combinations of subjects, from the federal state of Baden-Wuerttemberg in Germany and analyzed the same. The results indicate that, other than user motivation, three areas, namely “regulations and specifications,” “technological infrastructure,” and “heterogeneity of students and teachers,” affect the adoption of digital tools. The Covid-19 pandemic, which inevitably led teachers to embrace digital tools, positively influenced the perception and immediate usefulness of digital tools. We assert that no other variable would have been able to universally influence technology usage and acceptance to such an extent as to replicate the findings of our study and simultaneously highlight the uniqueness of the current situation and the necessity for examining its impact.

## Background: Digitalization of education and Covid-19

The digitalization of society has brought about many new challenges for education in the twenty-first century. While various strategies for dealing with these rapid changes exist globally, we concede that digital tools are no longer dispensable in our daily lives (Ferrari, 2012; OECD, 2019). These changes have also affected education systems, as schools, teachers, and students alike are compelled to (further) develop their digital competencies in this regard (European Commission, 2020). International studies indicate that large differences exist across as well as within various countries in terms of the level of digitalization of schools as well as the digital competencies of students (Eickelmann, 2019; OECD, 2020b). It is therefore not surprising that the variety of starting points globally led to a variety of response mechanisms in reaction to the pandemic. In mid-March 2020, school were ordered to close in many countries as a relevant containment measure owing to the rising infection rates (Viner et al., 2020). By the beginning of May, the United Nations Educational, Scientific and Cultural Organization (UNESCO) Institute for Statistics reported country-wide school closures in 182 countries, which impacted nearly 1.3 billion students worldwide (OECD, 2020a). Based on data from the 2018 PISA study, Ikeda (2020) reported significant discrepancies regarding the preparedness of schools across OECD countries with respect to carrying out remote education. According to the report, on average, only half of the schools were able to offer an online learning management platform (LMP) and only two out of every three students had been attending schools in which teachers had the necessary technical and pedagogical skills to “integrate digital devices in instruction and effective resources to incorporate technology in digital or distance learning” (Ikeda, 2020, p. 2). For example, Germany underperformed in both of the examined attributes: Only one third of the schools reported to have an effective LMP, and only 40% of the schools reported having the professional resources required for their teachers to learn how to use digital devices.

Scientific studies examining the developments in the digitalization of education have often focused on the obstacles for introducing digital tools within schools, distinguishing between both external (infrastructure, policy) and internal (leadership, teachers) barriers on various levels. With the outbreak of the pandemic, however, teachers no longer had the liberty to choose whether to incorporate digital tools into their teaching, as the circumstances made this inevitable. Research has revealed great differences in the capabilities and attitudes of teachers with regard to accepting and using digital tools in their teaching process (Granić & Marangunić, 2019). Teachers are central in this new environment and are especially affected by it, which makes their experiences particularly interesting and relevant. We aim to better understand how this unique situation has affected the acceptance of (and experience with) digital tools in the context of distance teaching.

Therefore, we examine the different experiences that secondary school teachers from Germany had regarding the use and acceptance of digital tools under these unique circumstances based on theoretical considerations of the technology acceptance model (TAM) (Davis, 1986). Our findings contribute to this special issue in three ways: First, we offer a national perspective for Germany, providing information regarding both the development of the situation and how teachers in this country handled the situation. Second, due to the considerable impact of school closures and the nationwide introduction of distance learning across all age groups, we re-evaluate the interaction between the variables of user motivation to provide insights regarding the acceptance and use of technology in distance teaching. We use the widely recognized TAM for better understanding the influence of external factors on the multitude of experiences that teachers (and students) across the globe had (and still have) with respect to accepting and using digital tools for distance teaching and learning. Finally, we highlight the impact of Covid-19 as a unique and universal factor in promoting the digitalization of education and suggest and discuss its direct influence upon teachers’ acceptance and usage of digital tools in teaching.

## Framework: Technology Acceptance Model (TAM)

**Fig. 1:**
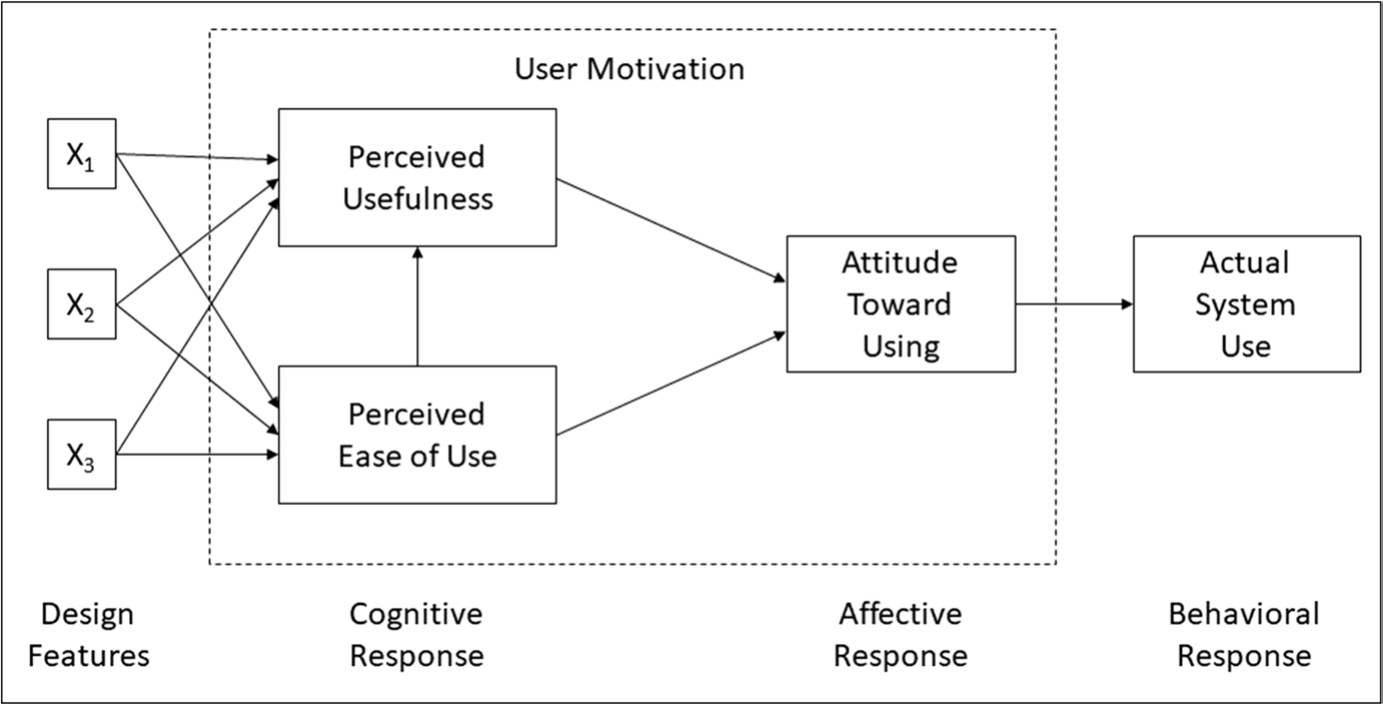
Technology acceptance model (Davis, 1986, p. 24)

Our study is based on a refined version (e.g. Sánchez & Hueros, 2010; Teo, 2011) of the well-established TAM developed by Davis (1986). The model is centered around established psychological theories, such as the theory of reasoned action, developed by Fishbein (1980), and the theory of predictable behavior described by Ajzen (1985). The TAM consists of three areas, which themselves comprise at least one variable. The core of the model (Fig. 1) consists of the variables of “perceived usefulness” (PU) and “perceived ease-of-use” (PEOU). More specifically, PU is said to indicate whether a user can make their task more efficient by means of technology. In the context of schooling, this would be whether a teacher believes that the use of digital tools allows them to conduct their lessons more efficiently. According to Teo et al. (2008), this can produce a noticeable difference, for example, by increasing efficiency during lesson preparation or during the individual lesson phases. PEOU, meanwhile, describes whether the user believes that applying a digital tool is effortless. In the school context, this would be represented as whether digital tools can be easily integrated into the classroom. This variable is usually highlighted by its absence, when the users of a system realize that its usefulness is overshadowed by the effort required to use it. In addition, the original model describes the variable “attitude toward using” (ATU) as a direct product of the two aforementioned variables while explaining a user’s motivation for adopting a certain technology. This third variable, ATU, for digital tools in the context of education, refers to the aversion or affection toward the use of digital tools in the classroom. While the first two variables can be assigned to different cognitive processes, according to Bresler (2016), the attitude towards digital tools is the direct outcome of emotional reactions. Thus, Davis (1986) assumes that cognitive processes of users determine their emotional attitude.

Notwithstanding, the PU and PEOU variables fail to fully explain the motives of users (Davis, 1986). This is due to the influence of external factors that determine user acceptance. These variables have been illustrated as “design features” in Fig. 1 on the left side and must be explicitly defined for each study. According to Taylor and Todd (1995), the significant external variables include subjective standards (perception of how important the use of technology is to other people) or self-efficacy (one’s own ability to deal with technology). Venkatesh and Davis (2000) further extend the model (referenced as TAM2) to include social influence processes (i.e. subjective norm, voluntariness, and image) and cognitive instrumental processes (i.e. job relevance, output quality, result demonstrability). The study also highlights discrepancies between voluntary and mandatory settings of technology implementation. Many further authors discuss in detail the interaction and relevance of considering further external variables (Burton-Jones & Hubona, 2006; Lee et al., 2003; Winarto, 2011). Finally, the model defines “behavioral response” as its result variable in terms of behavioral intent (BI) and use of technology (USE). Studies have shown that the non-tangible BI does not automatically lead to the tangible USE and that this is further influenced by other (external) variables (Bresler, 2016; Scherer et al., 2019).

### Technology acceptance amongst teachers

The rapid development of information systems and technology created the need to further define, validate, and refine theoretical models for understanding the acceptance and use of digital technologies in educational settings. Reviewing the research on the TAM conducted by Granić and Marangunić (2019) offers a broad overview of the current level of knowledge on TAM-Studies within the educational field. The authors conclude that the TAM has emerged as “a leading scientific paradigm for investigating acceptance of learning technology by students, teachers and other stakeholders” (p. 2575). From their discussion of the model and its underlying assertions, studies on technology acceptance are mostly based on self-reported usage of technology rather than actual usage. Furthermore, the studies were often limited to the inclusion of only one information system at a time, thus limiting the possibility of generalizing and comparing the data. Previous research has also focused on quantitative, questionnaire-based analyses. With regard to content, recent studies have focused on the relevance and (direct) influence of a multitude of external factors in association with the BI and USE, which resulted in various extensions and modifications of the original model. These modified frameworks (TAM2 or TAM + +) report increased interaction between the core variables (PEOU, PU, ATU) and external factors (i.e. self-efficacy, university support, social factors, etc.).

Research other than TAM-specific studies has analyzed various variables and factors that affect the interactions between teachers and digital tools. A crisis-induced change, as discussed by Lockton and Fargason (2019), for example, forces many teachers to confront their identity as teachers, their social convictions, or the norms followed in their school. According to the aforementioned authors, the willingness of teachers to change and a general positive attitude toward digital tools both affect the integration of digital tools into the teaching process. Understanding how these digital tools benefit teachers and learners is a key area of research (Bingimlas, 2009). According to Hatlevik (2017), the concept of self-efficacy plays an important role in this context, as it enables the distinction between the teachers’ confidence regarding the private use of digital tools and the appropriate use of the same in a vocational pedagogical context. Hammond et al. (2011) find that teachers who present low self-efficacy with respect to handling digital tools are among those whose usage of digital tools in class is the least.

Furthermore, teachers often report a shortage of time as being an obstacle to using digital tools in the classroom. Interestingly, according to Schmid et al. (2017), teachers spend a great deal of time searching for free, tested, and systematized study materials, especially open educational resources, and only share their personal materials, in analog form, with selected colleagues. Additionally, a majority of teachers are convinced that the USE of digital tools in class utilizes too much time and, thus, prefer to use analog media (Al Mulhim, 2014). Many teachers also feel that their workload is too high to attend training courses for digital tools in addition to their schoolwork, as this time is not counted, or only partially counted, as a part of their working hours. Moreover, Guo et al. (2008) reported that age has no significant influence on the use of and attitude toward digital tools.

Finally, the associated infrastructure was found to affect the acceptance and usage of media in teaching. For instance, Schmid et al. (2017) report that almost half of all teachers complain about the technical equipment in German schools (including poor Wi-Fi coverage and inadequate IT support). In addition, there is also a deficiency in the learning opportunities and training for teachers in the field with respect to digitalization and digital literacy (Waffner, 2020).

### Research gap: Technology acceptance in times of Covid-19

With regard to the recent modifications and state of research on TAM +  + , we argue that the closure of schools and the transition to distance teaching and remote learning due to the Covid-19 pandemic can be considered external factors that directly influence the usage and acceptance of digital tools in teaching. Teachers (and students alike) were forced into a unique situation in which the application of digital tools was unavoidable. To obtain a better understanding of the acceptance and usage of digital tools in distance teaching, we employ an extended TAM and knowledge from previous research to conduct interviews with teachers from Germany and to analyze the same. No studies have been conducted on the subject in the country to date (Granić & Marangunić, 2019). Our study begins from this point and examines the following overarching research question:

- Bullet Which factors influence teachers with regard to accepting and using digital tools for distance teaching during the Covid-19 pandemic?

## Method

To answer our research question, we conducted an interview study in Germany, more specifically, in the federal province of Baden-Wuerttemberg. Here, the federal government suspended all school activities beginning on March 16^th^ 2020, which was initially supposed to last for one month—till the end of the Easter vacation. Due to this decree, teachers were required to produce appropriate learning content and transmit this to students in order to enable distance learning (Ministry of Culture, Youth and Sport of Baden-Wuerttemberg, 2020). However, in actuality, schools in Baden-Wuerttemberg remained closed for nearly three months, re-opening for smaller groups of students in mid-June. We wanted to capture the situation without delay or falsification caused by the dynamics involved with remembered experience over time (Becker et al., 2002). Thus, we conducted interviews during this unique time with the teachers of secondary schools who were coerced into undertaking distance teaching using digital tools.

### Instrument

The findings of this study are based on a qualitative data analysis of 15 interviews. We developed a semi-structured interview guide based on the reviewed literature on the TAM with our main research question being the focus. The semi-structured interview format allows for a detailed understanding of topics and social settings and provides a certain degree of flexibility in the interview process based on the background, experience, and status of the interviewees (Denzin & Lincoln, 2011). The interview guide consisted of eight questions that related to the main variables of the model. In addition, we used a short questionnaire to obtain the socio-demographic information of the participants. Two interviewers conducted the interviews in May and June 2020 via videoconferences using the software Skype, BigBlueButton, and Microsoft Teams. The interviews lasted between 29 and 66 min, were audio-recorded, and were transcribed verbatim according to a specific transcription guideline based on the work of Dresing und Pehl (2020). Non-verbal signals were deliberately omitted to improve the readability of the transcripts. In addition, the transcripts were subjected to a de facto anonymization to avoid the possibility of specific statements being traced back to individual teachers. We replaced not only personal characteristics but also personal details, such as institutions or brands, with pseudo-information. We generated 173 pages of single-spaced transcribed text, and the total interview time was 674 min.

### Participants

A purposeful sampling strategy based on the work of Patton (2015) was employed. Regarding the selection criteria for this study, it was determined that teachers from the federal state of Baden-Wuerttemberg who teach classes of the secondary levels I and II (with students who are ten to sixteen years old) would be included. In addition, the teachers were required to have different professional experience from each other and teach different combinations of subjects. Furthermore, we considered whether the teachers had different workloads associated with extra-curricular activities at their school, such as departmental management or collaboration on the media development plan commission, as well as different familial responsibilities. An overview of the teachers interviewed has been summarized in Table 1. The letter in the pseudonym stands for the gender of the teacher, while the number represents the order in which the interviews were conducted.

**Table 1:** Participants (sorted by gender)

Pseudonym	Subjects Taught	Teaching experience (in years)	Age	Teaching load (in hours)*	Children
M01	Music, Physical Education (P.E.), Maths	14	48	25	2
M02	Biology, Geography, Ethics, Science & Technology	15	43	25	2
M03	Maths & P.E	2	35	12,5	2
M04	Maths & Geography	16	45	25	2
M05	Maths & P.E	1	29	25	0
M06	German, History, Social Studies	6	36	22	0
M07	Spanish, History, Social Studies	6	36	24	0
M08	Maths & P.E	1	29	25	0
M09	Chemistry, Biology, Science & Technology,	6	36	25	2
W01	Maths, Biology, Computer Science, Science & Technology	3	29	12,5	0
W02	German & Geography	26	58	22	0
W03	Biology, Chemistry, Science & Technology	11	39	16	2
W04	Biology & Maths	0	26	20	0
W05	Physics, Maths, Science & Technology	0	28	23	0
W06	P.E. & German	9	38	8	3

*A full teaching load consists of 25 h/week

### Data analysis

We performed a qualitative content analysis based on the study of Mayring (2015) for the 15 interview transcripts. The authors read the transcripts repeatedly and coded specific segments using MAXQDA Analytics Pro 2020 as per the deductive categories found through the literature review (e.g. perceived usefulness, tools applied, infrastructure, etc.) as well as the inductive categories that emerged from the transcribed interview material (e.g. professional development, maladministration, extracurricular obligations, etc.). The qualitative data analysis resulted in 29 codes and 1.168 coded segments.

We extracted quotes from the interview transcripts to illustrate the findings and interpretations related to our research question. All the interviews were conducted in German, and the lead author translated the quotes into English. We then critically reflected on the translations to ensure that the “voice” of the participants was maintained and that possible misunderstandings were avoided (Denzin & Lincoln, 2011).

## Findings and discussion

In this section, we present the findings obtained through our interviews with the teachers regarding their experiences with using digital tools for teaching during the first weeks of the Covid-19 pandemic. These have been divided into two sections: First, we present and discuss user motivation (PEOU, PU, ATU) in terms of how it affects technology acceptance based on the TAM (Davis, 1986). Second, we illustrate and discuss further (external) factors that affected the acceptance and usage of digital tools in our sample.

### User motivation

All interviewees were forced to use digital tools in the first weeks of the schools being closed due to the pandemic. Their individual experiences with these were, however, extremely diverse. While most teachers used digital tools for distance teaching, there were also teachers who used little or no digital tools for their lessons. This can be illustrated by the example of teacher W03, who used analog worksheets or task lists containing textbook tasks to provide her students with learning materials.

The teachers who integrated digital tools into their online classes often used LMPs, such as Microsoft Teams (M03, W04), BigBlueButton, or Moodle (M01), video conference tools, such as Skype, Jitsi (M01), or Zoom (M03), educational videos (M02), or messenger apps, such as WebUntis (M03, M05). The use of digital data acquisition systems (W05) was less common, and other apps were used via tablets (W01) as well.

For communicating with students and parents, the teachers reportedly relied on e-mails, messenger services, and/or their personal phone. As there was no official *modus operandi,* differences existed in how well this worked in practicality. This was explained by W05 as follows:

There are still teachers who are simply not really accessible digitally. And that is difficult for the parents; but in the end […] a large number of them [succeeded].

This heterogeneity between teachers is further exacerbated by the divergent understandings of what is considered to be a “digital tool” and how it is integrated into the teaching process. In the following sections, we will present and discuss our findings based on the PU, PEOU, and ATU variables of digital tools.

#### Perceived usefulness

Most of the interviewees conceded that digital tools were useful for staying in contact with students, distributing teaching materials, and trying to conduct lessons given the unique circumstances. Only one teacher (W03) vehemently maintained that she did not see any benefit in the application of digital tools outside of the absolute minimum requirements. Altogether, most teachers were quite optimistic about the new opportunities available to them. The choice of tool or environment seemed to be quite fundamental for the understanding of the usefulness of the same. Teachers spoke of the benefits of having one LMP that has various functions with which they were able to directly communicate with entire classes, individual students, colleagues, and parents, upload material and further links, correct homework and worksheets, organize a schedule, and keep track of attendance. The perceived usefulness often took precedence over any doubts and knowledge regarding the gray areas related to data protection and privacy.

Being coerced to utilize digital tools due to the pandemic motivated most teachers to reconsider the role of such tools in teaching for both distance learning and future lessons:

“It [digital teaching] works really well and […] I’m actually looking forward to the time ahead. So, of course, when things get back to normal at some point, I’ll say, ‘Wow, now I can actually incorporate this stuff into my teaching.’ Because now everyone knows how it works in theory.” (W05)

While previous studies have highlighted the challenges involved in introducing digital tools in teaching (Cheung & Huang, 2005; Lockton & Fargason, 2019; Schmid et al., 2017), the teachers in our sample tended to focus on the positive aspects related to the usefulness of specific tools (mostly as communication tools). Notwithstanding this development, according to the TAM, the perceived usefulness must be considered in direct association with the pandemic and closure of schools as an external factor. Our findings highlight the opportunities for replacing face-to-face teaching with digital tools during this unusual time. Analogously, the teachers emphasized that this can only be considered a transition under these peculiar circumstances and that they would not replace traditional teaching with the current form of distance learning. One teacher concludes the following:

“For the mere provision of materials, […] when we just look at what content we want to teach in school, so to speak, I don’t see any problem with digital tools at all. All of these process-related competencies and personal competencies, [on the other hand,] I don’t think we are able to depict digitally. Or [at least] not to the same extent as we could in the classroom.” (W01)

We discussed this issue with the teachers to gain a better understanding of the perceived opportunities associated with digital tools for traditional teaching and received inspiring responses from them. These covered topics such as the sustainability of digital board notes, reduction of stationery costs, doing justice to the heterogeneity of students, developing students’ digital literacy, catering to students’ interests, etc., to name but a few. While these ideas inspire hope, we cannot surmise positive, sustainable development in terms of the acceptance or usage of digital tools in teaching in the future based on our findings. Previous research has shown that teachers adapt to change slowly and tentatively (Lockton & Fargason, 2019). Therefore, if the PU is reduced to the aspects of communication (as seems to be the case in our sample), we believe that there will be little change in the acceptance and usage of digital tools over time.

#### Perceived ease-of-use

Most teachers reported that, once acquainted with the newly introduced tools, they realized it was not “rocket science” (W05). Although many interviewees were skeptical at the beginning, they seemed to adapt quickly and showed a willingness to learn. There is a large divide, however, between the PEOU for the use of digital tools as a communication tool (e.g. learning platforms, video conferences, e-mails) and that for use as a teaching tool (e.g. specific apps, learning videos).

Those participants who focused on using digital tools for communication reported to be more reserved toward applying digital tools for teaching and acknowledged that they would not have tried or used these tools if not for the ongoing crisis. This was accompanied by the reliance on spousal (W03) or collegial (M06) assistance, “several rookie mistakes” (M09), and frustration (M07). On a positive note, most of these challenges were overcome quite quickly, and all the teachers were able to handle and use digital tools for communication to carry out distance teaching within the first weeks, as exemplified by W02:

[…] a colleague briefed me a bit on Moodle and gave me a few tips. And I had a very nice colleague who was always online. So, I could always reach him. And he also gave me little tips and corrected me and so on. But it actually happened relatively quickly. I think, after a week, I had Moodle up and running. And since then, I’ve been doing everything online.

The more technologically informed participants were able to quickly and easily adapt to using digital tools as the new method of communicating with their students. Some of the teachers had already been using digital tools as convenient communication tools within their teaching process and were therefore at ease with this transition to distance teaching. It was interesting to note that these teachers wanted to further develop and refine their digital literacy and began familiarizing themselves with more profound aspects of digital tools, such as the creation of educational videos (M05, M08) or the integration of content-specific apps (M01, W01). These teachers were grateful for both the extra time and the endorsement for trying out new tools.

Our interviews highlight that the PEOU cannot be operationalized identically for all teachers, as their understanding of digital tools is as diverse as their self-efficacy regarding computers. As in other studies, these findings highlight the effect of external factors and their correlation with the PEOU variable (Gong & Xu, 2004; Hatlevik, 2017). Further, we contend that those teachers who were more anxious about adopting digital tools, due to their alleged complexity, did so nonetheless. However, we highlight again that this was solely because of the pandemic (as an external factor) and is by no means transferable to other situations. In the aftermath of Covid-19, however, we hope that this positive attitude toward the PEOU will translate into stronger self-confidence for teachers with regard to using digital tools, thereby increasing USE.

#### Attitude toward using digital tools

We wanted to acquire a better understanding of the overall ATU of our sample for using digital tools (in both personal life and teaching). Most of the respondents were found to be quite positive in their perception of digital tools, independent of the perceived PEOU and PU. The teachers critically reflected on the (personal) use of digital tools, and most accepted that these were a part of our daily lives in the twenty-first century. Mostly, the perception of digital tools was of a practical nature, as exemplified by the following quote from M02:

For me, digital tools are tools that I want to master, that are meant to benefit me. But I am not the slave of the digital tools I use.

One teacher’s ATU proved to be quite negative, which was based on her fear of not understanding the tools and not wanting to have someone explain everything “a hundred times over” (W03). Other teachers were found to be a bit resigned with respect to digital tools. This was mostly due to their previous negative experiences of such tools being extremely time consuming and then “simply not working” (W02). As the original TAM acknowledges that ATU influences USE directly and significantly, we suspect that the pandemic, as an external factor, overrules this variable and positively influences the usage of digital tools due to the necessity of distance teaching. The USE, in turn, seems to positively influence the acceptance of digital tools: Although the introduction and integration of (new) digital tools turned out to be quite resource intensive, the interviewees were reportedly grateful for this push (M01). In general, we maintain that a positive ATU was beneficial for a more advanced integration of digital tools in this phase of distance education (Abdullah et al., 2016).

Analogously, we also identified technophile teachers who were critical of the use of digital tools in the classroom:

“We [need] nothing more than […] good friends and recognition. Or, if you say it a bit poetically, the love of other people. And I don’t think you can convey that via digital tools. And there, this might actually be a barrier.” (M08)

Hereby, this teacher alludes to the spaces for bodily transcendence defined by Silverstone (2002), which counteract a corporeality in which we feign a constant presence, thus creating the new barriers of social status and data protection.

### External factors that influence the acceptance and usage of digital tools in distance teaching

We identified various external factors that directly and indirectly impacted the intent to use and the actual usage of digital tools as well as the extent to which teachers integrated these in the context of distance learning. Additionally, we examined the unique implications of the Covid-19 pandemic as a crucial external factor for USE (independent of the other core variables within the TAM). With these findings, we add to previous research that has focused on the significance of external factors for technology acceptance (Bresler, 2016; Granić & Marangunić, 2019; Scherer et al., 2019) and offer unique insights during this peculiar time of crisis. We have structured our findings in four sections, focusing on three identified external factors (regulations, infrastructure, and heterogeneity of students and teachers) as well as on the direct implications created by Covid-19.

#### Regulations and specifications

We found that national and federal regulations and specifications (as well as the lack thereof) affected the usage of digital tools amongst some of the participants. This supports the claims made by Venkatesh and Davis (2000) to distinguish between mandatory and voluntary usage settings. The official closing of schools and the transition to distance learning served as the trigger for the integration of some form of digital format for schools and teachers. During the first weeks of schools being closed, however, the Federal Government of Germany suspended all classes as well as events at school (Ministry of Culture, Youth and Sport of Baden-Wuerttemberg, 2020). This made teaching in general and using digital tools quite challenging:

“Because grading of student performance was officially suspended […]. In other words, some students knew exactly that if they do not send me anything, essentially nothing will happen. Except that I will be in a bad mood and grumble around. That means I never really received anything from all the students of a class.” (M06)

At the same time, other teachers regarded this as an opportunity to try new things and learn more about digital tools that can be used in teaching (M09, W01), following the motto “All is optional, nothing is a must.” Teacher M08, among others, was overwhelmed by this sudden change in the working process, as he felt it did not allow for the uniform teaching of all students.

Reflecting on the first weeks after schools were closed, the participants recalled missing the interactions with their students (M01, M09, W04) and colleagues (M06). It is interesting to note that the teachers did not discuss the risks associated with the virus and the risk of infection at all. They did not actually comment on the decision to close schools and accepted it as a necessary byproduct of the pandemic. One interviewee described how she enjoyed this change in her daily routine:

“ I [enjoy] this freedom, which allows for much more flexibility in your daily planning. This way, it is a lot easier to say, now is a good time for a break.” (W01)

On the other hand, some teachers complained about the lack of structure and clear requirements (M09) and the constantly changing information regarding the development of the pandemic and the status of school closure (M08).

Accepting the closing of schools as given, the actual usage of the digital tools was affected by whether and how the regulations and specifications regarding the implementation of distance learning were communicated. Most teachers spoke positively about the support and specifications that they received from their respective school (see Abdullah et al., 2016). The aspect of leadership has proven to be formative in the digitalization processes of education (Pettersson, 2018) and was repeatedly mentioned in the interviews. Internal communication needed to be adapted for digital communication, which served as a positive example for the overall communication with students, colleagues, and parents (M06). However, this exemplary communication was not always successful, as explained by M09:

[…] sometimes I would have liked a clearer statement from the school leadership, because in normal life I can’t say, ‘No, I won’t do it now.’ Then the boss would say, ‘We’re using this system now, get used to it!’ So, it’s absurd that it’s even possible as an official (to say), ‘No, I won’t do it. I’m not entering my grade into this weird digital system.’ But it is—it's a service directive, period! So, yes, I would sometimes like to have a bit more assertive.

This was also the case concerning data privacy and security—a topic that most teachers mentioned. M09 concluded the following:

Of course, it’s quite pleasant that the current crisis has triggered a bit of ‘Lalala, we’re not watching, do what you want’ among some people, so that you could test some new things.

As data privacy has been an important and widely discussed issue in the German society since the General Data Protection Regulation of the EU in 2018, the teachers were extremely aware of certain boundaries. At the same time, the topic of data privacy is very diffused, and, rather than crossing boundaries, the teachers tended to go out of their way to avoid these altogether (M05). In this sense, we conclude that data privacy was sometimes used as an *excuse* to justify a lack of integration of digital tools in past and current teaching (M03, W02). We understand the issue about data protection and the lack of transparency to be a strong barrier with respect to technology integration in both past and future teaching. M02 summarized this as a critique toward the government:

We always have a problem with data protection. It [The data] now runs via Microsoft servers—Are we allowed to store internal school information there or not? […] If there is no [federal state] server that works, then you have to switch to another one. And, at the moment, unfortunately, they [the government] haven’t managed to offer us a central, statewide option that works equally well. Of course, Moodle is now available on state servers. But all of these capacities are exhausted. Therefore, it’s just not comparable.

Overall, the teachers in our study chose their own individual paths, either ignoring data privacy altogether (intentionally or out of ignorance) or justifying their lack of technology integration with issues related to data privacy.

#### Technological infrastructure

The substantial amount of existing infrastructure has made technology usage quite diverse, as M02 explained, “It just can’t be right that a school doesn’t have laptops or iPads or anything for its teachers.” While some teachers reported being very well equipped (M05), others had to buy new devices to be able to teach from home (W02). When asked about what would further enhance the integration of digital tools in the future, most teachers focused a fully functional infrastructure for schools, students, and teachers alike.

Additionally, several of the interviewed teachers reported facing difficulties in installing and acquainting themselves with the new programs (M01, W02) and relied on or preferred the support from their spouse or colleagues. Previous studies have highlighted the positive influence of technical support and its influence on PU and PEOU and have described an increase of motivation related to the usage of technologies (Lim & Khine, 2006; Sánchez & Hueros, 2010). For those teachers in our sample who struggled with digital tools, it was this (technical) support that served as one of the most significant external factors in the acceptance and use of (the bare minimum of) digital tools. The deployment or appointment of a specific teacher/colleague for technical support was praised and highlighted repeatedly. The unbureaucratic accessibility to support at short notice enabled several teachers to implement their distance teaching via learning management tools. The interviewees used various mediums of communication to seek the support of these colleagues, such as telephone, e-mail, skype, and face-to-face interactions. Furthermore, several teachers highlighted the provision of tutorial videos, created by these “support colleagues” for the sole purpose of empowering teachers to implement the digital tools needed for distance teaching (M09, W01).

These findings substantiate previous studies that have highlighted the lack of access to hardware as well as the use of unsuitable software as challenges for integrating digital tools in lessons (Al Mulhim, 2014). The lack of infrastructure and support for technical equipment in German schools has repeatedly been documented in international studies (Eickelmann et al., 2019; Schmid et al., 2017). Notwithstanding, we find the process of confirming this to be highly problematic and preposterous in a wealthy country such as Germany.

#### Heterogeneity of students and teachers

Furthermore, teachers recognized the diversity among students and their respective situation at home in terms of support (M04, M08), available infrastructure (M03, W04), and insufficient knowledge (M02, M07) as limiting factors for integrating digital tools. Although all teachers recognized these differences, only a few considered these in their implementation of distance learning. One teacher, M06, explained how he reacted to these differences:

[…] I did not create any materials for which you have to use any particular device. […] That is, I made sure not to actually use anything that students who lack certain technical requirements are not able to work with.

The findings further highlighted the impact of personal differences as a factor that influenced technology acceptance. Rather than trying to define a certain type of teacher who accepts or rejects technology, we found certain factors that influence their attitude to varying degrees and seem to be dependent on other variables. On the one hand, we found professional expertise to affect the experience that teachers had with distance learning during the initial weeks. Our findings follow the presumption proposed by Fransson et al. (2019) that a large pool of analog material negatively influences technology acceptance. Hence, teachers who had such a large pool of analog material were more reluctant to adopt newer forms of technology and relied on digitized versions of the same material (W05), whereas M03 reported the following:

And then I try, I would say, to do lessons with it (distance learning) as normally as possible. In normal lessons, I also use the textbook as the basics. I always have other tasks from worksheets, and I try to set other impulses via videos or other media. And I tried to continue this in the [video conferences].

Another interesting finding was that several teachers reported their own subject as being unsuitable for distance learning or integrating with digital tools (W03, W06). The same teachers had preconceived ideas regarding how the integration of digital tools in other subjects could maybe work (better). The teacher W01, in turn, justified her positive attitude toward digital tools by linking this to the subjects she teaches.

The participants described the closure of schools as a pivotal moment in their acceptance of the pandemic being real (M06). They were optimistic that the situation would not last long and that schools would re-open after the Easter holidays. As time went by, the daily routine of the teachers changed. While most worked from home from the beginning, some preferred to initially work from their schools and switched to working from home a few weeks later (M06). For some teachers, the daily routine changed only marginally, while others struggled to find a new rhythm:

“Well, for school, it was always necessary for me to leave the house at half past six in the morning. Now, […] [I] get up more at seven or half past seven. So this daily rhythm has adjusted a bit, shifted a bit. Even the end of the day. At school, it was more common to get off at half past four, five, maybe even four o’clock. That has now also shifted back a bit.” (W01)

Several interviewees spoke positively about this change in their daily routine and about not having to commute to school every morning. Although this did save some time on a daily basis, the teachers reported the schedule for distance learning to be quite turbulent and unsteady, as explained by M04:

“Peace and quiet did not come at all. In other words, contrary to what some would have thought, that the teachers can rest for now. It wasn’t quite like that, if at all, then at first, maybe a little at the beginning.” (M04)

Finally, our study demonstrated that time constraints (i.e. family responsibilities with children at home during lockdown and reduced workloads) had a negative effect on the commitment to integrating digital tools into lessons. The participants with children, who would normally have been in school, spoke about the challenges of combining their familial commitments and their professional responsibilities. This finding is not surprising, as studies have shown that the integration of digital tools requires time-consuming preparation (especially in the initial phase) (Schmid et al., 2017). This setting was more challenging for the female participants (W03, W06) than for the male participants with similar familial responsibilities (M01, M03). Here, the stereotypical, traditional role distributions seem to appear.

#### Impact of Covid-19

The acceptance and usage of technology, according to the original TAM, is directly influenced by user motivation, which, in turn, is influenced by a variety of external factors (Davis, 1986). While previous research has examined and discussed the multitude of influential external factors, as well as their interaction with each other, these have consistently linked with user motivation (Gong & Xu, 2004; Granić & Marangunić, 2019; Lee et al., 2003; Venkatesh & Davis, 2000). The findings of our study indicate that considering the pandemic as an external factor does not fit the logic of this model.

Instead, we suggest that the impact of Covid-19 on technology is universal and influences USE directly, analogous to mandatory settings in TAM2 (Venkatesh & Davis, 2000). This is the case for user motivation, operationalized by the PU, PEOU, and ATU. With regard to the PU, the teachers in our sample perceived an immediate added value by using digital tools mostly as a medium that enabled communication with students, colleagues, and parents. Without the pandemic, this would not have been absolutely necessary and even inconceivable for some teachers. The PEOU and ATU were both also directly influenced by the immediacy of the pandemic. Our findings further reveal that most teachers were grateful to have been forced to deal with digital tools and were positively surprised by their usability (refer Section 4.1.2). We suspect (and hope) that the pandemic could lead to a greater openness amongst teachers toward the use of digital tools in education in the near future. The included external factors were also directly impacted by the pandemic: being crucial for the pronounced regulations and specifications (refer Section 4.2.1) and highlighting the diversity of the technological infrastructure available to schools and teachers (refer Section 4.2.2). Though the pandemic does not directly influence personal factors associated with teachers and students (refer to Section 4.2.3), we suggest that the unique circumstances emphasized and amplified existing differences. Environmental factors, such as familial responsibilities, and the implications thereof became much more momentous.

The previous presentation and discussion of our findings proposes that the pandemic has forced teachers to accept and use digital tools for teaching and has simultaneously directly influenced both user motivation and external factors. We assert that no other variable is capable of influencing USE to such an extent as to replicate the findings of our study. Thus, we highlight the uniqueness of the current situation and emphasize the necessity to examine its impact.

## Limitations and outlook

In discussing and formulating hypotheses about our findings and suggesting the possible next steps, we also need to understand the limitations of the study and our interpretation of its findings. First, we contemplate what will happen when schools return to traditional teaching methods and whether this phase of distance teaching will have a sustainable impact on technology acceptance amongst teachers in the future. We suggest that the situation can have a positive sustainable impact if we build on the lessons learned during this time. While we expect there to be lasting effects on both the PEOU and ATU for specific digital tools, the PU of these might quickly disappear in the conventional classroom environment. The PU of digital tools for educational purposes needs to be made tangible and communicated to the teachers (e.g. within further education programs). While the user motivation of teachers can be influenced through further education, the identified external factors mainly relate to the political demands and macro-development of education.

Furthermore, we want to caution the reader with regard to deriving recommendations for action based on our small, regional sample of teachers. While we were extremely committed to identifying a heterogeneous sample, we cannot rule out a self-selection bias. As a result, the sample might portray a social bias in favor of teachers who have a generally positive attitude toward digital tools. This is because those teachers who are generally against the use of such tools might not be willing to participate in a study that focuses on their acceptance and usage. While we do not intend to formulate generalizations based on our findings for the overall population of Germany, much less globally, we maintain that our findings are highly interesting and relevant for international readership. While large political, cultural, and economic differences exist globally, the pandemic and the need for the digitalization of education is universal. We can and need to learn from each other. With this in mind, we believe it is vital to share and build a common knowledge base during these unusual circumstances.

Future studies should focus on the unique effects of Covid-19 on teaching and the digitalization of education. In order to understand the sustainability of the changes in the acceptance and usage of digital tools, we suggest the employment of longitudinal study designs over the next years. Additionally, quantitative surveys on technology acceptance in times of distance teaching could strengthen our hypothesis of the pandemic being a unique factor in impacting technology acceptance and usage in education.

## Conclusion

In this study, we examined the unique effects of Covid-19 on the factors that influenced teachers’ acceptance and usage of digital tools for distance teaching. For this purpose, we conducted 15 semi-structured interviews with secondary school teachers and analyzed the transcripts based on the theoretical framework of the TAM.

We found that the user motivation amongst the interviewees during this unique time was generally positive. We suggest that this is largely dependent on the impact of Covid-19 and distance teaching and might change with the return of face-to-face teaching. Furthermore, we found that the PEOU influences not only the teachers’ acceptance of tools but also their type of usage; teachers with previous experience tested and integrated more advanced digital tools (e.g. learning videos) than those who were confronted with these for the first time (mostly for the purpose of communication).

With regard to external factors, we identified regulations and specifications, technological infrastructure, and the heterogeneity of the students and teachers to affect teachers’ acceptance and usage of digital tools. Again, we would like to highlight the universal impact of Covid-19 on all the variables of the model and suggest that, contrary to previous literature, the pandemic, considered an external factor, directly influences USE.

We are optimistic that 2020 can be a game-changer in the digitalization of education and believe that teachers have the potential to make that change happen. Understanding the factors that foster or deter teachers’ acceptance and usage of digital tools for teaching is a vital move(ment) toward supporting them in this process. With our study, we have taken the first explorative step and encourage further studies to build upon our findings.
